# Effects of Ergonomics Training Plus Structured Exercise Versus Ergonomics Training Alone on Musculoskeletal Pain, Disability, and Burnout in Schoolteachers: A Randomized Controlled Trial

**DOI:** 10.3390/healthcare14213813

**Published:** 2026-11-09

**Authors:** Muharem Akar, Burak Buğday, Suzan Aydın, Ömer Şevgin

**Affiliations:** 1 Department of Physiotherapy and Rehabilitation, Faculty of Health Sciences, Üsküdar University, 34662 Istanbul, Türkiye; muharemakar34@gmail.com; 2 Department of Gerontology, Faculty of Health Sciences, İnönü University, 44280 Malatya, Türkiye; burak.bugday@inonu.edu.tr; 3 Department of Physiotherapy and Rehabilitation, Faculty of Health Sciences, Istanbul Gelişim University, 34310 Istanbul, Türkiye; suaydin@gelisim.edu.tr

**Keywords:** teachers, musculoskeletal disorders, exercise therapy, ergonomics, neck pain, low back pain, burnout, randomized controlled trial

## Abstract

**Background/Objectives**: Musculoskeletal disorders are common among teachers, but randomized evidence on adding exercise to ergonomics training is scarce. This trial compared ergonomics training plus structured exercise with ergonomics training alone. **Methods**: Eighty teachers with musculoskeletal pain lasting at least three months were stratified by school level and randomized 1:1. Both groups attended four ergonomics sessions and received weekly telephone calls; the ergonomics-plus-exercise group also performed a 12-week, largely self-directed program of stretching, strengthening, and core stabilization. Registered primary outcomes were the Oswestry Disability Index (ODI), Neck Disability Index (NDI), Maslach Burnout Inventory–Educators Survey, and Nordic Musculoskeletal Questionnaire; pain intensity (VAS) was secondary. A blinded assessor collected outcomes at baseline and week 12. **Results**: All participants completed the trial. Group × time interactions favored ergonomics plus exercise for ODI (difference in change −6.80; 95% CI −9.22 to −4.38), NDI (−4.90; −7.53 to −2.27), emotional exhaustion (−2.63; −3.89 to −1.36), depersonalization, and personal accomplishment (Holm-adjusted *p* ≤ 0.013), and for VAS (−1.18; −1.48 to −0.87). At least 30% improvement occurred in 80% versus 10% of participants for ODI and 90% versus 38% for NDI, but in 95% versus 93% for VAS. The number of symptomatic body regions decreased more with exercise (−0.67; −1.15 to −0.20); in unadjusted exploratory region-specific analyses, only the shoulder reached nominal significance, with baseline imbalance. **Conclusions**: Adding a structured exercise program to ergonomics training gave additional short-term improvements in disability and burnout. Mean differences were modest, outcomes and adherence were self-reported, and participants knew their allocation.

## 1. Introduction

Musculoskeletal disorders are a leading cause of disability worldwide [1,2], and teachers are among the occupational groups in which they are common. A meta-analysis of 44 studies covering nearly 16,000 teachers reported an overall prevalence of 68%, with the neck (47%), low back (47%), and shoulders (44%) most often affected [3]. This figure is of the same order as the roughly three in five workers who report musculoskeletal complaints in the European workforce as a whole [4], and it approaches the 77% annual prevalence pooled across nurses [5]. Teachers therefore carry a burden close to that of occupations with heavy manual handling, although their work involves little lifting. Similarly high figures have been reported in studies of teachers from Africa, Latin America, and Türkiye [6,7,8,9].

The exposure profile of teaching explains this pattern. Teaching involves prolonged standing, sustained forward head posture while marking and preparing lessons, repeated arm elevation when writing on the board, and extended computer use [10]. These symptoms are associated with poorer quality of life [10,11]. They are more frequent in physical education teachers than in classroom teachers [12] and are highly prevalent in special education teachers [13]. In one survey, nearly half of teachers reported absenteeism due to neck or shoulder pain or low back pain [14]. The burden is not only physical: musculoskeletal pain and burnout often occur together in schoolteachers, with workload related to exhaustion [15], and physical activity appears to be related to burnout risk in teachers, although the relationship depends on the type and context of the activity [16].

Ergonomics training is the most common preventive response. It is an educational intervention that teaches workers to recognize and reduce the physical risk factors in their own work: adjusting the workstation and its tools, adopting neutral postures, using safer techniques for recurrent tasks, and scheduling breaks. Unlike exercise therapy, manual therapy, or medication, it is directed at the exposure and not at the physical capacity of the worker or at the symptoms. Its effect on pain is small when results are pooled across occupations [17]. In teachers, an educational ergonomics program was followed by a lower prevalence of neck, shoulder, and back pain, but the evidence comes from an uncontrolled pre–post study [18].

There are reasons to expect that exercise adds to ergonomics training. Much of the load in teaching cannot be removed by ergonomic adjustment, because standing, board work, and marking are inherent to the job. Exercise acts on the other side of the balance by increasing the strength, endurance, and mobility with which the remaining load is tolerated. Exercise is supported by systematic reviews for chronic neck pain [19] and chronic low back pain [20], and it probably lowers the risk of new neck pain episodes in working populations [21]. Teacher-specific trials are few. Adding deep cervical flexor training to conventional exercise reduced neck pain and disability in schoolteachers [22], a three-week at-work exercise program added to an ergonomic brochure reduced low back and neck pain in nursery school teachers [23], and workplace yoga improved pain in female teachers when compared with no intervention [24]. Part of the rationale therefore rests on trials in other occupations with largely static work, such as office workers [25].

Whether exercise gives a benefit beyond ergonomics training itself is less clear. An integrative review suggested that combined ergonomic and physical activity interventions may reduce neck and hand or wrist pain in workers [26]. In randomized trials that used ergonomics training as the comparator, added exercise reduced pain more in tea workers [27] and factory workers [28] but not in construction workers [29]; a pilot trial in hemodialysis nurses found less discomfort after a combined program [30], and in office workers, stretching combined with ergonomic modification was no better than either alone [31]. In teachers, the only trial of this kind lasted three weeks and did not assess burnout [23]. To our knowledge, no randomized trial in teachers has tested a longer exercise program against ergonomics training while assessing both physical outcomes and burnout.

This trial therefore asked whether adding a 12-week structured exercise program to ergonomics training improves low back and neck disability, burnout, musculoskeletal symptoms, and pain intensity in schoolteachers with musculoskeletal pain, compared with ergonomics training alone. We hypothesized that the combined program would produce greater improvements. An exploratory aim was to describe symptoms and burnout by school level.

## 2. Materials and Methods

### 2.1. Study Design, Ethics, and Registration

This was a two-arm, parallel-group randomized controlled trial with a 1:1 allocation ratio and blinded outcome assessment, reported in line with the CONSORT 2025 statement [32]. It was conducted in schools affiliated with the Tatvan District Directorate of National Education (Bitlis, Türkiye). The Üsküdar University Non-Interventional Research Ethics Committee approved the protocol (No. 61351342/020-2008; meeting of 25 February 2026), and the District Directorate granted permission to conduct the study. The study followed the Declaration of Helsinki [33], and all participants gave written informed consent. No payment or other incentive was offered for participation or completion.

The trial was registered prospectively at ClinicalTrials.gov (NCT07442383; https://clinicaltrials.gov/study/NCT07442383 (accessed on 3 November 2026)). The record was submitted on 25 February 2026 and posted on 2 March 2026. The first participant was enrolled on 10 April 2026, and recruitment continued until early June 2026. Each participant was followed for 12 weeks, and the last week-12 assessment was made on 30 August 2026. Differences between the registration and this report are listed in Section 2.9.

### 2.2. Participants and Setting

Teachers working at preschool, primary, middle, and high school levels were invited to participate. Inclusion criteria were: age 30 to 60 years; at least five years of teaching experience; musculoskeletal pain lasting at least three months; pain intensity of at least 3 on a 0–10 Visual Analog Scale; and ability to attend the training and exercise program. Exclusion criteria were: a serious neurological or orthopedic disease; musculoskeletal surgery within the previous six months; and any medical or psychological condition that would prevent exercise.

Participants were recruited from 13 schools, with one to seven participants per school. Teachers of both groups worked in the same schools. To limit contamination, the ergonomics training and the exercise instruction were delivered individually, and teachers in the ergonomics-plus-exercise group were asked not to share the exercise program with colleagues.

### 2.3. Sample Size

The sample size was calculated a priori with G*Power 3.1 [34] (F tests; ANOVA: repeated measures, within–between interaction). The inputs were an effect size f of 0.25, α of 0.05, power of 0.95, two groups, two measurements, a correlation of 0.5 between repeated measures, and a nonsphericity correction of 1, which gave a minimum total sample of 54. The effect size was the conventional medium value for the group × time interaction and was not derived from a specific outcome or from pilot data. The target was raised to 80 teachers to allow equal representation of the four school levels (20 per level, 10 per group) and to protect against attrition.

### 2.4. Randomization, Allocation Concealment, and Blinding

Randomization was stratified by school level. Before recruitment, a secretary who was independent of the research team prepared the allocation list for each stratum in Microsoft Excel: a computer-generated random number was assigned to each of the 20 sequence numbers of the stratum, the sequence numbers were sorted by this random number, and the first 10 were allocated to one group and the remaining 10 to the other. No blocks were used; the fixed 10:10 split within each stratum produced the balance. The list was kept by the secretary and was not accessible to the investigators. The physiotherapist who later delivered the interventions enrolled the participants and obtained consent, and a second physiotherapist performed the baseline assessment. Only then did the secretary disclose the assignment of that participant, in the order of enrollment within the stratum, face to face to the treating physiotherapist and the participant.

Participants and the treating physiotherapist were aware of the allocation. The assessing physiotherapist and the person who ran the statistical analysis were not. All questionnaires were administered as face-to-face interviews by the assessing physiotherapist, who read the items and recorded the answers, at baseline and at week 12. Because every outcome was reported by participants who knew their group, assessor blinding protects against biased recording but not against expectation or reporting bias.

### 2.5. Interventions

**Ergonomics training and telephone contact (both groups).** All participants attended four face-to-face sessions at school in weeks 1, 4, 8, and 12. The sessions were delivered individually, lasted 30 to 45 min, and combined a short explanation with practical demonstration. They covered classroom workstation ergonomics, posture awareness, the ergonomics of daily teaching tasks, and break strategies (Table 1). Participants received a workstation checklist, a brochure, and a weekly self-monitoring form. The fourth session introduced a 2 to 3 min set of micro-exercises for use between lessons, which consisted of shortened versions of the phase 1 neck and trunk stretches. This set was taught in week 12, at the end of the intervention period, so the ergonomics-only group received no exercise prescription before the final assessment. All 80 participants attended all four sessions. The treating physiotherapist also telephoned every participant in both groups once a week to ask about their general condition and about any adverse event. Participants in both groups were asked not to start any other treatment for their musculoskeletal complaints during the trial.

**Exercise program (ergonomics-plus-exercise group only).** This group also followed a 12-week program performed three times a week, a frequency used in an earlier workplace stretching trial [31], for a total of 36 sessions of about 45 min. The program had three 4-week phases (Table 1). Phase 1 consisted of stretching and postural awareness exercises. Phase 2 added scapular, isometric neck, and trunk extensor strengthening. Phase 3 added core stabilization, which improves pain and function in chronic non-specific low back pain [35]. The exercises of the earlier phases were continued in the later phases. Each session began with 2 to 3 min of light warm-up and ended with 1 to 2 min of diaphragmatic breathing.

Intensity was low to moderate. Stretches were held at the point of mild tension, isometric neck contractions were performed against light to moderate manual resistance, and strengthening and stabilization exercises used body weight only. Participants were instructed to work within a pain-free range and to stop any exercise that increased their pain. Progression was time-based: all participants moved to the next phase after four weeks, with the repetitions, sets, and holding times shown in Table 1. The program was standardized and was not tailored to individuals, and no exercise had to be modified or withdrawn for any participant.

The program was not supervised. The treating physiotherapist taught the exercises in one individual face-to-face session and gave each participant an illustrated written guide. Participants then performed the sessions on their own at school or at home. During later contacts, the physiotherapist occasionally checked technique and answered questions, but technique was not re-assessed on a fixed schedule. In the weekly telephone call, participants in this group were additionally asked how many of the three scheduled sessions they had completed. No exercise diary was kept, and session duration and the reasons for missed sessions were not systematically recorded. Adherence is therefore the self-reported completion of scheduled sessions.

Adverse events were defined as any new injury or any increase in pain that the participant attributed to the exercises or the training, and they were asked about in the weekly telephone calls in both groups. Compliance with the request to avoid other treatment, and leisure-time exercise outside the program, were not systematically recorded.

### 2.6. Outcome Measures

Outcomes were assessed at baseline (week 0) and after the intervention (week 12). Age, sex, school level, height, and weight were recorded at baseline.

**Low back disability (registered primary outcome).** The Turkish version of the Oswestry Disability Index (ODI) was used [36]. It contains 10 items on pain and daily activities and is expressed as a percentage from 0 to 100, with higher scores indicating greater disability.

**Neck disability (registered primary outcome).** The Turkish version of the Neck Disability Index (NDI) was used [37]. It contains 10 items and is expressed as a percentage from 0 to 100, with higher scores indicating greater disability. Both indices were administered to all participants, because neck symptoms (73 of 80, 91.3%) and low back symptoms (70 of 80, 87.5%) were highly prevalent and most teachers had symptoms in several regions. Sensitivity analyses were restricted to participants with symptoms in the corresponding region at baseline.

**Burnout (registered primary outcome).** The Turkish adaptation of the Maslach Burnout Inventory–Educators Survey (MBI-ES) was used [38]. Its 22 items were rated from 1 to 5 and form three subscales: emotional exhaustion (9 items; range 9–45), depersonalization (5 items; range 5–25), and personal accomplishment (8 items; range 8–40). Higher emotional exhaustion and depersonalization scores and lower personal accomplishment scores indicate greater burnout.

**Musculoskeletal symptoms (registered primary outcome).** The Turkish version of the Nordic Musculoskeletal Questionnaire (NMQ) [39] records, for nine body regions, whether the participant had trouble (ache, pain, or discomfort) in the previous 12 months and in the previous 7 days, and whether the trouble had prevented normal activity (at home or away from home) in the previous 12 months. Symptoms in the previous 7 days were used to evaluate change, because this recall period was the same at both assessments and did not overlap the pre-intervention period at week 12. A symptom was classed as resolved when it was present in the previous 7 days at baseline and absent at week 12, and as new when it was absent at baseline and present at week 12. The activity-limitation item (“Have you at any time during the last 12 months been prevented from doing your normal work because of the trouble?”) was administered in the same form at both assessments, but its 12-month recall period at week 12 overlaps the pre-intervention period, so a change in this item cannot be attributed to the intervention. Responses to this item were therefore not used to evaluate the intervention and are not reported.

**Pain intensity (secondary outcome).** Participants rated their current pain in the region that they identified as most painful at baseline, on a 10 cm Visual Analog Scale (VAS) anchored at 0 (no pain) and 10 (unbearable pain). The same region was rated at week 12. Scores were recorded to the nearest whole number.

### 2.7. Statistical Analysis

The original analyses were performed with SPSS version 22 (IBM Corp., Armonk, NY, USA). The additional analyses requested during peer review were performed on the same dataset with Python 3.13 (SciPy 1.18). All 80 randomized participants completed both assessments and were analyzed in the group to which they were allocated, so the intention-to-treat and complete-case populations are identical and no outcome data were imputed. Normality was assessed with the Kolmogorov–Smirnov test together with skewness and kurtosis values. Continuous variables are presented as mean ± standard deviation and categorical variables as *n* (%). Baseline characteristics are presented descriptively.

The registered primary outcomes were the ODI, the NDI, the three MBI-ES subscales, and the NMQ; the VAS was a secondary outcome. For each continuous outcome, the intervention effect was tested with the group × time interaction of a 2 × 2 mixed-design ANOVA, which with two time points is equivalent to comparing change scores between groups. Results are reported as the between-group difference in change with its 95% confidence interval, and partial eta squared (ηp^2^) with its 90% confidence interval [40]. To control the family-wise error rate across the registered primary outcomes, the Holm procedure was applied to one family of six tests: the five continuous primary outcomes and the questionnaire-wide NMQ analysis (the change in the number of symptomatic regions, described below). As sensitivity analyses, week-12 scores were compared between groups with adjustment for the baseline score (ANCOVA), and change scores were compared with the Mann–Whitney U test. Within-group changes (paired *t*-tests with Cohen’s dz) are given in Supplementary Table S1 for completeness and were not used to judge the intervention effect.

The registration did not specify how the NMQ would be summarized. Within-group change in the 7-day prevalence of symptoms in each region was tested with the exact McNemar test. Three between-group comparisons were then made: the change in the number of body regions with symptoms (independent-samples *t*-test, confirmed with the Mann–Whitney U test), which gives one test for the questionnaire as a whole and was included in the Holm adjustment of the primary outcomes; the proportion of baseline symptoms that had resolved at week 12 in each region; and the week-12 prevalence in each region. Differences in proportions are reported with Newcombe 95% confidence intervals and Fisher’s exact test. The region-specific comparisons were not adjusted for multiplicity and are exploratory.

Two further sets of analyses were not prespecified and are exploratory. First, the proportions of participants whose VAS, ODI, and NDI scores improved by at least 30% from baseline, a threshold proposed for clinically important change [41], were compared between groups; participants with a baseline score of zero were counted as non-responders. Second, school levels were compared with both groups pooled (one-way ANOVA with Bonferroni post hoc tests; Fisher–Freeman–Halton exact test for symptoms), and the group × school-level interaction on change scores was examined with two-way ANOVA. The significance level was *p* < 0.05.

### 2.8. Data and Materials

A separate statistical analysis plan was not written; the analysis followed the ethics-approved protocol and the description above. The exercise guide and the ergonomics brochure are available from the corresponding author on reasonable request. Participant-level data are not shared because of ethical and privacy restrictions (see Data Availability Statement).

### 2.9. Differences from the Trial Registration

The following differences between the registry record and this report were identified. (1) The VAS was an outcome in the ethics-approved protocol, which predates the registration, but it was omitted from the registry entry by oversight. It is reported here as a secondary outcome. (2) The registry gave a time frame of 14 weeks for the outcomes; this was an entry error, and the final assessment was made at week 12, at the end of the program. (3) The registry described masking as double. The masked roles were the outcome assessor and the data analyst; participants and the treating physiotherapist were not masked. (4) The registry listed a pain score below 3 as an exclusion criterion and a psychological disorder that prevents exercise as a further exclusion criterion; these correspond to the inclusion threshold of at least 3 and to the exclusion of conditions preventing exercise in Section 2.2. (5) The registry described the NMQ recall periods as 12 months, four weeks, and the day of assessment, whereas the questionnaire used in the trial asked about the previous 12 months and the previous 7 days. (6) The registry described MBI-ES items as scored from 0 to 4, whereas items were scored from 1 to 5; this shifts the subscale ranges but not the differences between groups. (7) The registry gave 15 July 2026 as the primary completion date; the last week-12 assessment was made on 30 August 2026. (8) The registry named the four instruments as primary outcomes without ranking them or defining an analysis for the NMQ; the hierarchy and the multiplicity adjustment described in Section 2.7 were defined during the analysis and peer review. The registry record has since been updated to correct these entries.

## 3. Results

### 3.1. Participants

**Figure 1:**
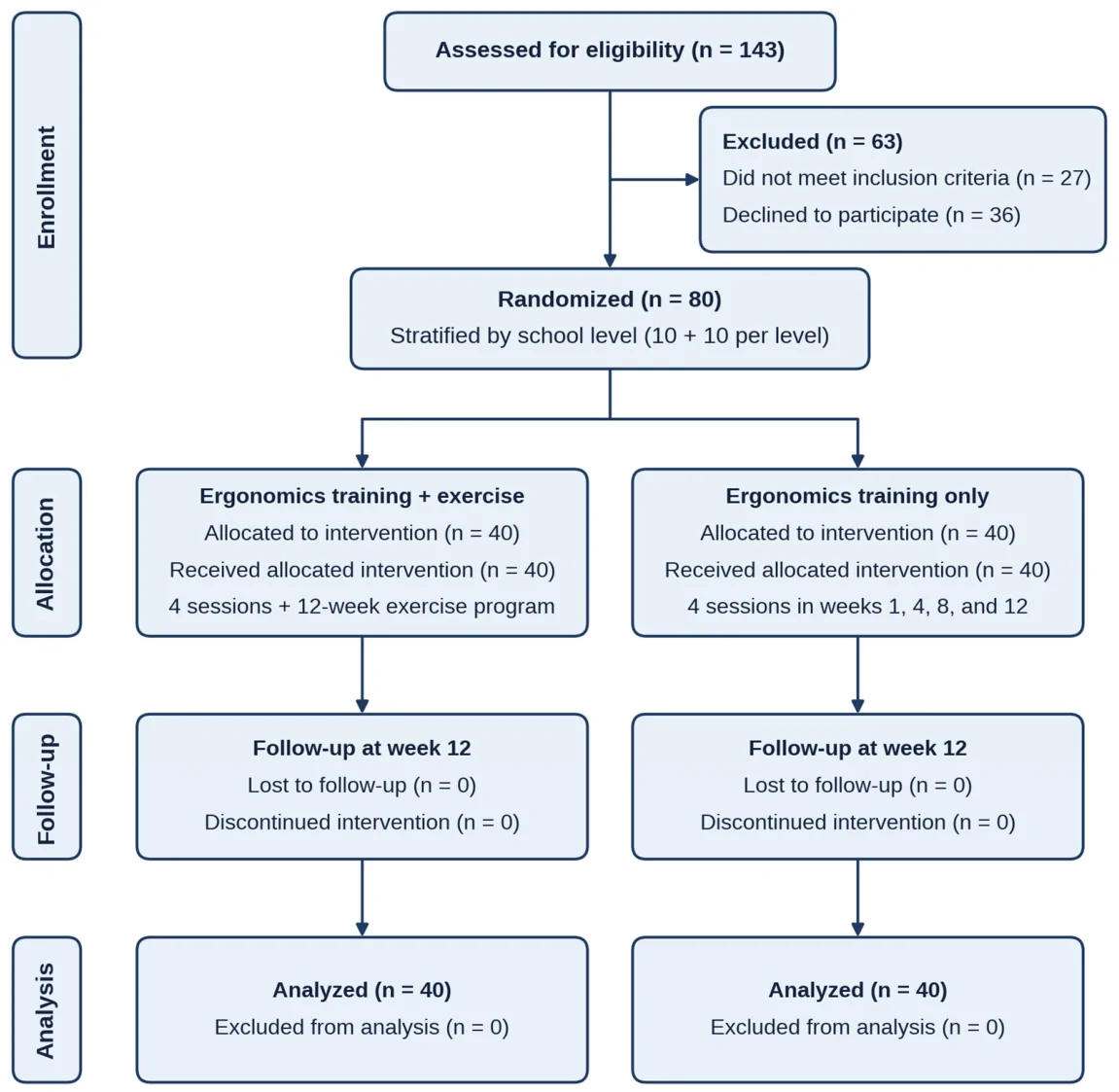
CONSORT flow diagram of the trial.

A total of 143 teachers were assessed for eligibility, and 63 were excluded: 27 did not meet the inclusion criteria and 36 declined to participate. Eighty teachers were randomized, 40 to each group, and all completed the 12-week period and the final assessment (Figure 1). Baseline characteristics are shown in Table 2. Shoulder symptoms in the previous 7 days were more frequent in the ergonomics-plus-exercise group (17 of 40) than in the ergonomics-only group (9 of 40); the other characteristics were similar.

### 3.2. Disability, Burnout, and Pain

**Figure 2:**
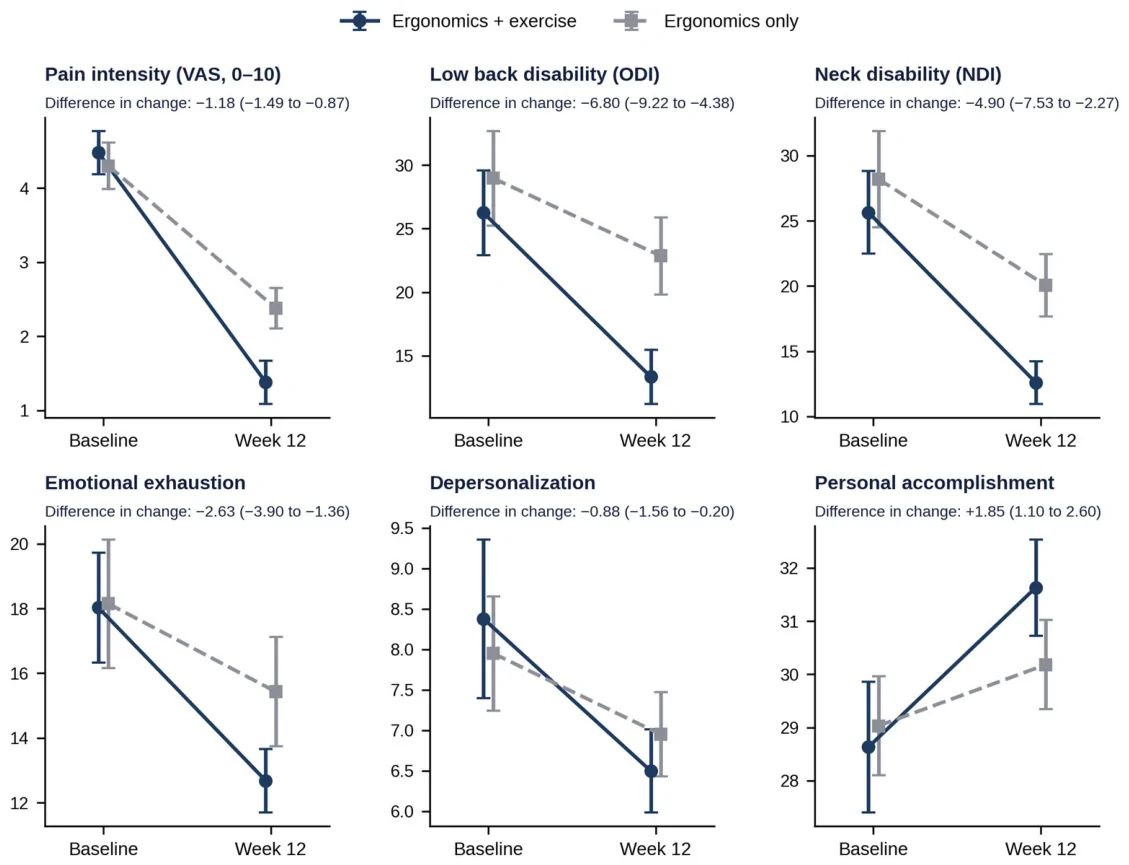
Mean scores at baseline and week 12 in each group. Error bars are 95% confidence intervals. The difference in change between groups, with its 95% confidence interval, is given in each panel. Lower scores are better for all outcomes except personal accomplishment.

The group × time interaction favored the ergonomics-plus-exercise group for all five continuous primary outcomes, and all remained significant after Holm adjustment across the six primary tests (Table 3, Figure 2). The between-group difference in change was −6.80 points for the ODI and −4.90 points for the NDI. For burnout, the differences were −2.63 points for emotional exhaustion, −0.88 for depersonalization, and +1.85 for personal accomplishment. Depersonalization showed the smallest effect (ηp^2^ = 0.08); it remained significant in the baseline-adjusted analysis but not in the Mann–Whitney comparison of change scores (*p* = 0.09; Supplementary Table S1). For the secondary outcome, the difference in change in VAS was −1.18 points. Baseline-adjusted analyses gave the same conclusions for all six outcomes (Supplementary Table S1).

In the exploratory responder analysis, an improvement of at least 30% from baseline was reached by 80.0% of the ergonomics-plus-exercise group and 10.0% of the ergonomics-only group for the ODI, and by 90.0% and 37.5% for the NDI (Table 4). For the VAS, almost all participants in both groups reached this threshold (95.0% and 92.5%), and the groups did not differ.

When the analysis was restricted to participants who reported symptoms in the corresponding region in the previous 7 days at baseline, the differences in change were −6.92 points for the ODI (95% CI −9.31 to −4.52; *n* = 70) and −5.36 points for the NDI (−7.82 to −2.89; *n* = 73), close to those in the full sample.

### 3.3. Musculoskeletal Symptoms

**Figure 3:**
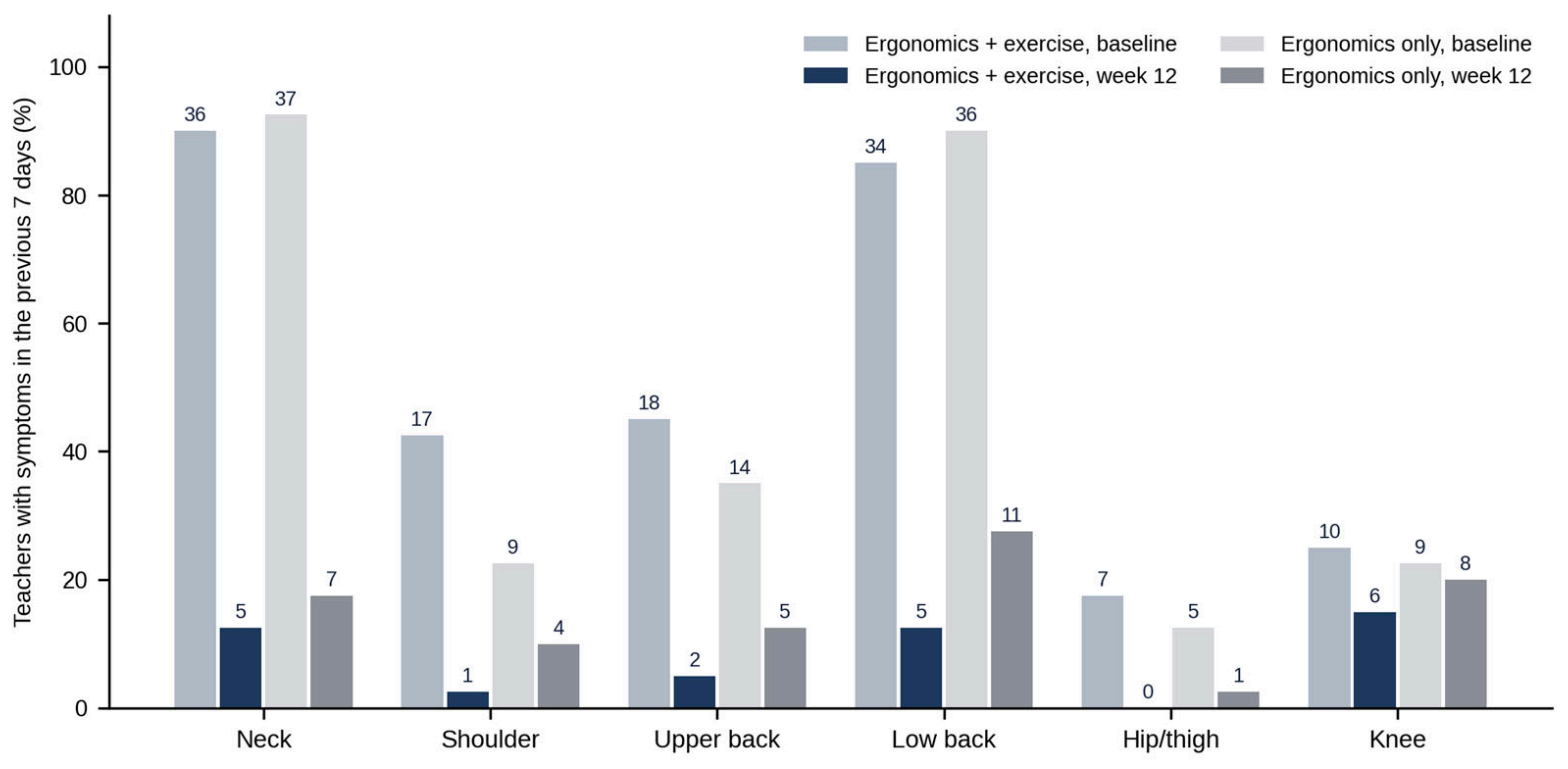
Teachers reporting symptoms in the previous 7 days at baseline and week 12, by body region and group. Numbers above the bars are counts (*n* = 40 per group).

At baseline, the neck and low back were the regions most often affected in the previous 7 days (Table 5, Figure 3). The mean number of regions with symptoms fell from 3.10 to 0.55 in the ergonomics-plus-exercise group and from 2.83 to 0.95 in the ergonomics-only group; the difference in change was −0.67 regions (95% CI −1.15 to −0.20; *p* = 0.006, Holm-adjusted *p* = 0.013; Mann–Whitney *p* = 0.008).

Within groups, the prevalence of neck, upper back, and low back symptoms decreased in both groups, and the prevalence of shoulder and hip or thigh symptoms decreased only in the ergonomics-plus-exercise group. In the unadjusted exploratory comparisons between groups, the proportion of baseline symptoms that had resolved was nominally higher with exercise only for the shoulder (16 of 17 versus 5 of 9; difference 38.6 percentage points, 95% CI 5.4 to 67.9; *p* = 0.034), a region in which the groups were unbalanced at baseline. Resolution did not differ significantly for the neck, upper back, low back, hip or thigh, or knee, and week-12 prevalence did not differ significantly in any region. New symptoms were rare (Table 5).

### 3.4. Exploratory Analyses by School Level

With both groups pooled, baseline emotional exhaustion and depersonalization were higher in middle and high school teachers than in preschool and primary school teachers, whereas baseline ODI, NDI, VAS, and personal accomplishment did not differ significantly by level (Supplementary Table S2). Knee symptoms in the previous 7 days were more frequent in high school teachers (11 of 20) than at the other levels (1 to 5 of 20; exact *p* = 0.001). The group × school-level interaction on change scores was significant for emotional exhaustion (*p* < 0.001) and depersonalization (*p* = 0.045) and not for the other outcomes; the difference between groups in emotional exhaustion was largest among high school teachers. With 10 teachers per group at each level, these analyses have low power and no multiplicity adjustment.

### 3.5. Adherence and Adverse Events

According to the weekly self-reports, 33 of the 40 teachers in the ergonomics-plus-exercise group (82.5%) completed all 36 scheduled sessions, 4 (10.0%) missed two sessions, and 3 (7.5%) missed one session, corresponding to 99.2% of scheduled sessions. These figures were not verified independently. All participants attended the four ergonomics sessions. No adverse events were reported in either group.

## 4. Discussion

In teachers with musculoskeletal pain, adding a 12-week structured exercise program to ergonomics training gave greater short-term improvements in low back and neck disability and in burnout than ergonomics training alone. The additional effect on pain intensity was statistically significant but small, and the questionnaire-wide reduction in symptomatic regions was greater with exercise, while in unadjusted exploratory region-specific analyses, only the shoulder comparison reached nominal significance, in the presence of baseline imbalance.

### 4.1. Size and Clinical Importance of the Effects

Statistical significance and clinical importance have to be separated. Proposed thresholds for a minimal important change in low back pain are 15 points on a 0–100 VAS, 10 points on the ODI, or an improvement of 30% from baseline [41]. The mean differences between groups, 1.18 VAS points (11.8 on a 0–100 scale) and 6.80 ODI points, were below the absolute thresholds, and the upper limits of their confidence intervals (1.48 VAS points and 9.22 ODI points) did not reach them. The average added benefit of the exercise package was therefore modest. This is expected with an active comparator: the ergonomics-only group itself improved by 1.9 VAS points and 6 to 8 points on the disability indices.

The responder analysis qualifies this picture. For pain, almost every participant in both groups improved by at least 30%, so exercise did not change the chance of a clinically relevant pain response. For disability, the proportion of responders was much higher with exercise, by 70 percentage points for the ODI and 53 for the NDI. This suggests that the main contribution of the exercise package was to function and not to pain. The responder analysis was not prespecified, the 30% threshold was developed for patients with low back pain, and some participants had low baseline scores, so a relative threshold can be reached with small absolute changes. These proportions should be read as supportive and exploratory.

### 4.2. Comparison with Previous Exercise Trials

The size of the added effect on pain is similar to that reported in the two earlier trials in teachers that used an active or minimal comparator. In schoolteachers with neck pain, adding deep cervical flexor training to conventional exercise for six weeks reduced pain by 2.0 points on a 0–10 scale compared with 0.9 points with conventional exercise alone, a difference of about 1.1 points [22]. In nursery school teachers, an extension-oriented exercise program added to an ergonomic brochure reduced low back pain by 1.7 points more than the brochure alone after two months [23]. The larger difference in the latter trial is consistent with its comparator, a brochure after which pain did not change, whereas our comparator was a four-session training after which pain fell by almost two points. Workplace yoga improved pain and disability in female teachers, but against no intervention [24]. Our result therefore agrees with the teacher literature and extends it to a 12-week program, a face-to-face ergonomics comparator, and burnout outcomes.

Trials with the same design in other occupations give a mixed picture. Exercise added to ergonomics training reduced pain more than ergonomics training alone in tea workers [27] and factory workers [28]. In construction workers, the adjusted difference in pain was 0.34 points and not significant, and burnout did not improve more with exercise [29]. In office workers, stretching combined with ergonomic modification was no better than either alone [31]. The present difference of 1.18 points lies between these results. A plausible explanation is the load profile. Construction work involves heavy dynamic loading that an exercise program of this intensity may not offset, whereas the load in teaching is dominated by static postures and prolonged standing, for which stretching, postural endurance, and trunk stabilization are directly relevant.

The content of the program follows the exercise types with the best support. Motor control exercise reduces chronic neck pain in the short term [19], core stabilization and related exercise improve pain and function in chronic low back pain [35,42], and exercise programs reduce pain and disability from work-related musculoskeletal disorders in manual workers [43]. A network meta-analysis found that three sessions a week and 12-week programs reduce low back pain, although sessions of 15 to 30 min and programs of 16 weeks or longer ranked highest [44]. The program was low in intensity, used no external load, and was unsupervised. Larger effects might be obtained with supervised or progressively loaded training, but at a higher cost and probably with lower adherence.

### 4.3. From Prevention to Recovery: What the Combination Adds

The two components of the program act at different points of the continuum from prevention to recovery. Ergonomics training is a preventive strategy: it addresses the occupational exposure by changing the workstation, the posture, and the organization of tasks, and its aim is to reduce the load that produces symptoms in the first place. Structured exercise is a rehabilitative strategy: it addresses the functional capacity of the worker who already has symptoms, by restoring mobility, strength, and endurance. The results reflect this division. The ergonomics-only group improved, which is consistent with a reduction in exposure, and the addition of exercise produced its largest additional effects on the disability indices, which measure function. A workplace program that combines the two therefore covers both the exposure and the capacity side of musculoskeletal health, whereas either alone leaves one side unaddressed.

The trial does not show that the combination is better than exercise alone, because it had no exercise-only arm. What it shows is that ergonomics training, which is the usual occupational response and was followed by clear improvement on its own, leaves room for further gains, mainly in function. The two components address different targets: the training reduces exposure, and the exercise raises the capacity to tolerate the exposure that remains. For school health services, the practical implication is that an ergonomics course alone may not be sufficient for teachers who already have symptoms. Whether exercise alone would achieve the same result, and whether the combination is cost-effective, cannot be answered with these data.

The intervention effect also has to be understood as the effect of a package. Both groups had the same four ergonomics sessions and the same weekly telephone calls, so the amount of contact was similar. The ergonomics-plus-exercise group additionally received an individual teaching session, an illustrated guide, questions about the exercises in the weekly call, and occasional checks of technique. The observed differences therefore combine the physiological effect of exercise with the effects of this instruction and monitoring and of the expectations of participants who knew that they were receiving the additional treatment. The design cannot separate these components.

### 4.4. Regional Musculoskeletal Symptoms

Neck and low back symptoms in the previous 7 days were reported by 85% to 93% of participants at baseline, which is higher than the pooled prevalence in teachers [3], because pain was an inclusion criterion. Symptoms decreased markedly in both groups. The formal comparison between groups supports a greater reduction in the number of symptomatic regions with exercise, by about two-thirds of a region on average. For individual regions, the evidence is weak: only the shoulder comparison reached nominal significance in unadjusted analyses, shoulder symptoms were twice as frequent in the exercise group at baseline, the confidence intervals are wide, and no adjustment was made for the number of regions tested. We therefore do not conclude that symptoms in any specific region resolve more often with exercise. Knee, elbow, wrist, and ankle symptoms were infrequent and did not change, which is plausible because the program contained no exercises for the extremities.

### 4.5. Burnout

All three burnout subscales improved more in the ergonomics-plus-exercise group, although the program had no psychological component. Less disability may reduce the daily effort that teaching requires and thus lessen emotional exhaustion, in line with the co-occurrence of musculoskeletal pain and burnout in teachers [15]. Physical activity is often associated with lower burnout, particularly lower emotional exhaustion and depersonalization, in health care workers [45]; in teachers, higher physical activity was associated with lower burnout in a cross-sectional study [46], and a prospective study found associations that varied with the intensity and domain of the activity [16].

These findings need particular caution. Both groups were telephoned every week, so the frequency of contact with the physiotherapist does not explain the difference. Burnout is nevertheless the outcome most open to expectation in participants who knew their allocation, and the exercise group may have perceived more support because its calls concerned a program that it was carrying out. The effect on depersonalization was small and was not significant in the non-parametric sensitivity analysis. A trial with the same comparator in construction workers found no additional benefit for burnout [29]. Although the MBI-ES was a registered primary outcome, the program did not target the psychological or organizational determinants of burnout, and we therefore interpret the burnout results as exploratory. They are best regarded as a hypothesis to be tested in a trial with blinded participants or an attention-matched sham exercise comparator.

### 4.6. School Level

Middle and high school teachers started with higher emotional exhaustion and depersonalization, and the group × level interaction suggested a larger difference between groups in emotional exhaustion at the upper levels. Larger changes in those with higher baseline values may reflect regression to the mean, each cell contained 10 teachers, and several interactions were tested. These analyses do not establish that the effect of the program differs by school level. They raise two hypotheses for future trials: that teachers at upper school levels with high burnout may respond more, and that programs for high school teachers, who often reported knee symptoms, may need lower-limb exercises.

### 4.7. Strengths and Limitations

Strengths of the trial are prospective registration, stratified randomization with a concealed allocation list held by an independent person, an active comparator with the same schedule of sessions and weekly telephone calls, balanced representation of four school levels, complete follow-up and full attendance, and interviewer-administered outcomes collected by an assessor unaware of allocation.

The limitations bear directly on the interpretation. First, all outcomes were reported by participants who knew their allocation, and the exercise group may have expected and reported more improvement; assessor blinding does not remove this bias, and no objective measures such as range of motion, strength, or posture were collected. The estimates may therefore overstate the true effect. Second, although contact was similar in the two groups, the effect belongs to the whole exercise package (instruction, guide, and exercise-specific monitoring) and not to exercise as such. Third, adherence was self-reported in weekly calls made by the treating physiotherapist, without diaries or objective monitoring. The reported completion of 99.2% of sessions is unusually high for a self-directed program and probably overestimates the true dose through recall and social desirability; technique was checked only occasionally, so the quality and intensity of the exercise performed are uncertain. Fourth, outcomes were measured only at week 12, immediately after the program, so nothing can be said about durability. Fifth, the trial was conducted in a single district with volunteers, which limits generalizability; teachers of both groups worked in the same schools, and although instruction was individual and participants were asked not to share the program, contamination cannot be excluded and would bias the difference toward zero. Sixth, the registration did not rank the outcomes and omitted the VAS, the responder, regional NMQ, and school-level analyses were exploratory, and co-interventions and leisure-time exercise were not systematically recorded. Finally, the VAS referred to the most painful region and not to a fixed anatomical site, so it is a measure of each participant’s main complaint.

### 4.8. Practical Implications

The program required one individual teaching session, an illustrated guide, no equipment, and a brief weekly call, and all participants completed the trial without reported adverse events. It is therefore feasible in schools and promising for the short-term management of disability in teachers with musculoskeletal symptoms. Higher adherence to workplace strength training has been associated with clinically relevant reductions in neck and shoulder pain [47], and booster sessions and supervision may improve adherence to physiotherapy exercise [48], so periodic contact is likely to be a necessary part of such a program. Recommendations for routine implementation should await replication in other regions, longer follow-up, objective outcomes and recorded adherence, a comparator that controls for expectation, and an evaluation of resources and cost-effectiveness.

## 5. Conclusions

In teachers with musculoskeletal pain, adding a 12-week structured exercise program to ergonomics training produced statistically significant additional short-term improvements in low back and neck disability, burnout, and pain intensity. The mean differences between groups were modest and, for pain and low back disability, below proposed thresholds for a minimal important change, although more participants reached a 30% improvement in disability. The number of symptomatic body regions decreased more with exercise, but differences for individual regions were not established. Because outcomes and adherence were self-reported, participants knew their allocation, and follow-up ended at week 12, the program should be regarded as a feasible strategy that supports further implementation studies rather than as an established recommendation; its routine use in school-based occupational health programs needs confirmation in trials with objective outcomes, verified adherence, control for expectation, longer follow-up, and cost evaluation.

## Figures and Tables

**Table 1:** Content of the ergonomics training and the exercise program.

Component	Timing	Content	Dose
Ergonomics session 1 (both groups)	Week 1	Classroom workstation: desk and board height, screen and book distance, spinal alignment when standing and sitting; workstation checklist	30–45 min
Ergonomics session 2 (both groups)	Week 4	Neutral spine, forward head posture, cumulative effect of static postures; mirror or partner feedback; posture check every 30 min	30–45 min
Ergonomics session 3 (both groups)	Week 8	Arm and shoulder position at the board, carrying materials, safe bending and lifting, weight shifting during prolonged standing	30–45 min
Ergonomics session 4 (both groups)	Week 12	Micro-breaks; a 2–3 min micro-exercise set (shortened phase 1 stretches) for use between lessons; self-monitoring for long-term maintenance	30–45 min
Weekly telephone call (both groups)	Weeks 1–12	General condition and adverse events; in the ergonomics-plus-exercise group, as well as the number of exercise sessions completed	Once a week
Exercise phase 1: stretching and postural awareness	Weeks 1–4	Chin tuck; neck lateral flexion, rotation, flexion–extension, and diagonal stretches; upper trapezius stretch; shoulder circles; seated thoracic extension; cat-cow; pelvic tilt	Stretches: 20 s × 2 per direction. Chin tuck and pelvic tilt: 10 reps × 2 sets, 5 s hold. Shoulder circles: 10 forward and 10 backward. Thoracic extension and cat-cow: 10 reps × 2 sets
Exercise phase 2: strengthening added	Weeks 5–8	Scapular retraction; multidirectional isometric neck training (flexion, extension, lateral flexion) against light to moderate manual resistance; prone back extension; bridge	Scapular retraction: 15 reps × 2 sets. Isometric neck: 10 reps × 2 sets per direction, 5 s hold. Back extension: 10 reps × 2 sets, 2–3 s hold. Bridge: 12 reps × 2 sets, 5 s hold
Exercise phase 3: core stabilization added	Weeks 9–12	Bird-dog; single-leg bridge	Bird-dog: 8–10 reps × 2 sets per side, 3–5 s hold. Single-leg bridge: 10 reps × 2 sets per leg
All exercise sessions	Weeks 1–12	Warm-up 2–3 min; exercises; diaphragmatic breathing 1–2 min; low-to-moderate intensity, body weight only; progression by phase every 4 weeks	About 45 min, 3 days a week, 36 sessions; self-directed after one individual teaching session

**Table 2:** Baseline characteristics of the participants.

Characteristic	Ergonomics + Exercise (*n* = 40)	Ergonomics Only (*n* = 40)
School level, *n* (%)		
Preschool	10 (25.0)	10 (25.0)
Primary school	10 (25.0)	10 (25.0)
Middle school	10 (25.0)	10 (25.0)
High school	10 (25.0)	10 (25.0)
Sex, *n* (%)		
Male	17 (42.5)	18 (45.0)
Female	23 (57.5)	22 (55.0)
Age (years)	36.75 ± 4.54	37.58 ± 5.26
Body mass index (kg/m^2^)	25.74 ± 2.68	26.47 ± 6.34
ODI	26.25 ± 10.56	28.95 ± 11.86
NDI	25.65 ± 10.01	28.20 ± 11.75
MBI-ES emotional exhaustion	18.03 ± 5.41	18.15 ± 6.32
MBI-ES depersonalization	8.38 ± 3.11	7.95 ± 2.24
MBI-ES personal accomplishment	28.63 ± 3.90	29.03 ± 2.96
VAS (0–10)	4.48 ± 0.93	4.30 ± 0.99
NMQ symptoms in the previous 7 days, *n* (%)		
Neck	36 (90.0)	37 (92.5)
Shoulder	17 (42.5)	9 (22.5)
Upper back	18 (45.0)	14 (35.0)
Low back	34 (85.0)	36 (90.0)
Hip/thigh	7 (17.5)	5 (12.5)
Knee	10 (25.0)	9 (22.5)
Number of symptomatic regions (0–9)	3.10 ± 1.06	2.83 ± 1.06

NMQ, Nordic Musculoskeletal Questionnaire; ODI, Oswestry Disability Index; NDI, Neck Disability Index; MBI-ES, Maslach Burnout Inventory–Educators Survey; VAS, Visual Analog Scale.

**Table 3:** Continuous outcomes at baseline and week 12 and between-group differences in change.

Outcome	Group	Baseline	Week 12	Change	Difference in Change (95% CI)	F	*p*	Holm *p*	ηp^2^ (90% CI)
ODI	Ergonomics + exercise	26.25 ± 10.56	13.35 ± 6.78	−12.90	−6.80 (−9.22 to −4.38)	31.32	<0.001	<0.001	0.29 (0.15 to 0.41)
	Ergonomics only	28.95 ± 11.86	22.85 ± 9.60	−6.10					
NDI	Ergonomics + exercise	25.65 ± 10.01	12.60 ± 5.21	−13.05	−4.90 (−7.53 to −2.27)	13.76	<0.001	0.001	0.15 (0.05 to 0.27)
	Ergonomics only	28.20 ± 11.75	20.05 ± 7.64	−8.15					
MBI-ES emotional exhaustion	Ergonomics + exercise	18.03 ± 5.41	12.68 ± 3.12	−5.35	−2.63 (−3.89 to −1.36)	17.03	<0.001	<0.001	0.18 (0.07 to 0.30)
	Ergonomics only	18.15 ± 6.32	15.43 ± 5.38	−2.73					
MBI-ES depersonalization	Ergonomics + exercise	8.38 ± 3.11	6.50 ± 1.63	−1.88	−0.88 (−1.55 to −0.20)	6.62	0.012	0.013	0.08 (0.01 to 0.18)
	Ergonomics only	7.95 ± 2.24	6.95 ± 1.65	−1.00					
MBI-ES personal accomplishment	Ergonomics + exercise	28.63 ± 3.90	31.63 ± 2.88	+3.00	+1.85 (1.10 to 2.60)	24.15	<0.001	<0.001	0.24 (0.11 to 0.36)
	Ergonomics only	29.03 ± 2.96	30.18 ± 2.66	+1.15					
VAS (0–10)	Ergonomics + exercise	4.48 ± 0.93	1.38 ± 0.93	−3.10	−1.18 (−1.48 to −0.87)	59.21	<0.001	—	0.43 (0.29 to 0.54)
	Ergonomics only	4.30 ± 0.99	2.38 ± 0.87	−1.93					

Values are mean ± SD. The difference in change is the change in the ergonomics-plus-exercise group minus the change in the ergonomics-only group; F is the group × time interaction (df = 1, 78). Holm-adjusted *p* values cover the six primary tests (the five continuous primary outcomes and the change in the number of symptomatic regions on the NMQ, Section 3.3); the VAS was a secondary outcome and its *p* value is unadjusted. ODI, Oswestry Disability Index; NDI, Neck Disability Index; MBI-ES, Maslach Burnout Inventory–Educators Survey; VAS, Visual Analog Scale; CI, confidence interval.

**Table 4:** Participants with an improvement of at least 30% from baseline (exploratory analysis).

Outcome	Ergonomics + Exercise, *n* (%)	Ergonomics Only, *n* (%)	Risk Difference (95% CI)	*p*
ODI	32 (80.0)	4 (10.0)	70.0 (50.3 to 81.3)	<0.001
NDI	36 (90.0)	15 (37.5)	52.5 (32.3 to 67.1)	<0.001
VAS	38 (95.0)	37 (92.5)	2.5 (−10.0 to 15.4)	1.000

Risk differences are in percentage points with Newcombe 95% confidence intervals; *p* values are from Fisher’s exact test. Participants with a baseline score of zero (ODI, *n* = 4; NDI, *n* = 2) were counted as non-responders.

**Table 5:** Symptoms in the previous 7 days on the Nordic Musculoskeletal Questionnaire: paired transitions and between-group comparison of resolution.

Region	Group	Baseline, *n* (%)	Week 12, *n* (%)	Persisted	Resolved	New	Within-Group *p*	Difference in Resolution (95% CI)	*p*
Neck	E + E	36 (90.0)	5 (12.5)	5	31/36	0	<0.001	5.0 (−12.5 to 22.2)	0.754
	E only	37 (92.5)	7 (17.5)	7	30/37	0	<0.001		
Shoulder	E + E	17 (42.5)	1 (2.5)	1	16/17	0	<0.001	38.6 (5.4 to 67.9)	0.034
	E only	9 (22.5)	4 (10.0)	4	5/9	0	0.063		
Upper back	E + E	18 (45.0)	2 (5.0)	1	17/18	1	<0.001	30.2 (2.2 to 56.1)	0.064
	E only	14 (35.0)	5 (12.5)	5	9/14	0	0.004		
Low back	E + E	34 (85.0)	5 (12.5)	5	29/34	0	<0.001	10.3 (−8.8 to 28.4)	0.374
	E only	36 (90.0)	11 (27.5)	9	27/36	2	<0.001		
Hip/thigh	E + E	7 (17.5)	0 (0.0)	0	7/7	0	0.016	20.0 (−19.0 to 62.4)	0.417
	E only	5 (12.5)	1 (2.5)	1	4/5	0	0.125		
Knee	E + E	10 (25.0)	6 (15.0)	6	4/10	0	0.125	17.8 (−22.2 to 50.6)	0.628
	E only	9 (22.5)	8 (20.0)	7	2/9	1	1.000		
Elbow	E + E	0 (0.0)	0 (0.0)	0	0	0	1.000	NA	NA
	E only	1 (2.5)	1 (2.5)	1	0/1	0	1.000		
Wrist/hand	E + E	1 (2.5)	2 (5.0)	0	1/1	2	1.000	NA	NA
	E only	0 (0.0)	0 (0.0)	0	0	0	1.000		
Ankle/foot	E + E	1 (2.5)	1 (2.5)	1	0/1	0	1.000	NA	NA
	E only	2 (5.0)	1 (2.5)	1	1/2	0	1.000		

E + E, ergonomics plus exercise (*n* = 40); E only, ergonomics only (*n* = 40). Persisted, symptom at both assessments; resolved, symptom at baseline only; new, symptom at week 12 only. Within-group *p* values are from the exact McNemar test. The difference in resolution is the proportion resolved among those with the symptom at baseline (E + E minus E only), in percentage points with Newcombe 95% confidence interval and Fisher’s exact test; it was not calculated when fewer than five participants in a group had the symptom at baseline. NA, not applicable.

## Data Availability

The data presented in this study are not available because of ethical and privacy restrictions: the participants did not consent to the sharing of their individual data outside the research team, and the small, single-district sample carries a risk of re-identification; participant-level data therefore cannot be provided. The exercise guide and the ergonomics brochure are available from the corresponding author on reasonable request.

## Supplementary Materials

The following supporting information can be downloaded at: https://www.mdpi.com/article/10.3390/healthcare14213813/s1. Table S1: Within-group changes and sensitivity analyses for the continuous outcomes; Table S2: Exploratory analyses by school level.
