# Supplementary material for: Effects of Ergonomics Training Plus Structured Exercise Versus Ergonomics Training Alone on Musculoskeletal Pain, Disability, and Burnout in Schoolteachers: A Randomized Controlled Trial
Source: Healthcare (Basel). 2026 Nov 9;14(21):3813. doi: 10.3390/healthcare14213813 (PMC13762614; doi:10.3390/healthcare14213813)
Supplement: Supplementary file 1 [file healthcare-14-03813-s001.zip › healthcare-4631281-supplementary.pdf]

Supplementary Materials

Effects of Ergonomics Training Plus Structured Exercise Versus Ergonomics Training Alone on Musculoskeletal Pain, Disability, and Burnout in Schoolteachers: A Randomized Controlled Trial

Table S1. Within-group changes and sensitivity analyses for the continuous outcomes.

Outcome	Group	Change, mean ± SD	Cohen's dz	Paired t-test p	Baseline-adjusted difference at week 12 (95% CI)	ANCOVA p	Mann–Whitney p (change scores)
ODI	Ergonomics + exercise	−12.90 ± 7.00	−1.84	<0.001	−7.73 (−9.46 to −6.00)	<0.001	<0.001
	Ergonomics only	−6.10 ± 3.17	−1.93	<0.001			
NDI	Ergonomics + exercise	−13.05 ± 6.06	−2.15	<0.001	−6.09 (−7.44 to −4.74)	<0.001	<0.001
	Ergonomics only	−8.15 ± 5.75	−1.42	<0.001			
MBI-ES emotional exhaustion	Ergonomics + exercise	−5.35 ± 3.75	−1.43	<0.001	−2.67 (−3.58 to −1.75)	<0.001	<0.001
	Ergonomics only	−2.73 ± 1.45	−1.88	<0.001			
MBI-ES depersonalization	Ergonomics + exercise	−1.88 ± 1.96	−0.95	<0.001	−0.67 (−1.04 to −0.31)	<0.001	0.092
	Ergonomics only	−1.00 ± 0.88	−1.14	<0.001			
MBI-ES personal accomplishment	Ergonomics + exercise	+3.00 ± 1.85	1.62	<0.001	+1.73 (1.13 to 2.33)	<0.001	<0.001
	Ergonomics only	+1.15 ± 1.49	0.77	<0.001			
VAS (0–10)	Ergonomics + exercise	−3.10 ± 0.78	−3.99	<0.001	−1.12 (−1.39 to −0.84)	<0.001	<0.001
	Ergonomics only	−1.93 ± 0.57	−3.36	<0.001			

The baseline-adjusted difference is the coefficient for group (ergonomics plus exercise minus ergonomics only) in a linear model of the week-12 score with the baseline score as covariate. Cohen's dz is the mean change divided by the standard deviation of the change; within-group statistics are descriptive and were not used to judge the intervention effect. ODI, Oswestry Disability Index; NDI, Neck Disability Index; MBI-ES, Maslach Burnout Inventory–Educators Survey; VAS, Visual Analog Scale.

Table S2. Exploratory analyses by school level.

Variable	Preschool (n = 20)	Primary (n = 20)	Middle (n = 20)	High (n = 20)	F (3, 76)	p	Group × level interaction p
ODI, baseline	23.40 ± 13.60	32.00 ± 10.62	30.50 ± 7.89	24.50 ± 10.36	3.14	0.030	
ODI, change	−10.70 ± 8.14	−11.00 ± 7.61	−9.50 ± 2.96	−6.80 ± 5.04	1.85	0.145	0.326
NDI, baseline	27.30 ± 12.04	25.30 ± 11.59	27.70 ± 9.16	27.40 ± 11.35	0.20	0.899	
NDI, change	−13.10 ± 7.33	−10.10 ± 7.15	−9.80 ± 5.39	−9.40 ± 5.07	1.43	0.239	0.787

MBI-ES emotional exhaustion, baseline	15.00 ± 4.33	13.65 ± 2.54	21.30 ± 5.03	22.40 ± 5.52	19.16	<0.001	
MBI-ES emotional exhaustion, change	−2.35 ± 1.60	−2.55 ± 1.57	−4.70 ± 1.84	−6.55 ± 4.43	11.24	<0.001	<0.001
MBI-ES depersonalization, baseline	7.45 ± 1.90	6.30 ± 1.53	9.60 ± 2.91	9.30 ± 2.87	8.64	<0.001	
MBI-ES depersonalization, change	−0.65 ± 0.88	−0.60 ± 0.88	−2.45 ± 1.82	−2.05 ± 1.61	9.76	<0.001	0.045
MBI-ES personal accomplishment, baseline	29.60 ± 3.65	28.80 ± 4.32	28.85 ± 2.72	28.05 ± 2.95	0.67	0.575	
MBI-ES personal accomplishment, change	+2.25 ± 2.24	+2.10 ± 1.94	+2.55 ± 1.79	+1.40 ± 1.57	1.31	0.277	0.714
VAS (0–10), baseline	4.55 ± 0.94	4.50 ± 1.05	4.20 ± 0.89	4.30 ± 0.98	0.58	0.629	
VAS (0–10), change	−2.70 ± 0.80	−2.35 ± 1.09	−2.60 ± 1.05	−2.40 ± 0.60	0.67	0.576	0.069

**Change by group**	**Preschool**	**Primary**	**Middle**	**High**
MBI-ES emotional exhaustion, ergonomics + exercise (n = 10 per level)	−2.30 ± 1.70	−3.60 ± 1.26	−5.80 ± 2.04	−9.70 ± 4.16
MBI-ES emotional exhaustion, ergonomics only (n = 10 per level)	−2.40 ± 1.58	−1.50 ± 1.08	−3.60 ± 0.52	−3.40 ± 1.43
MBI-ES depersonalization, ergonomics + exercise (n = 10 per level)	−0.80 ± 0.92	−0.60 ± 0.97	−3.50 ± 2.01	−2.60 ± 2.07
MBI-ES depersonalization, ergonomics only (n = 10 per level)	−0.50 ± 0.85	−0.60 ± 0.84	−1.40 ± 0.70	−1.50 ± 0.71
Knee symptoms in the previous 7 days at baseline, n (%)	5 (25.0)	2 (10.0)	1 (5.0)	11 (55.0)
Knee symptoms in the previous 7 days at week 12, n (%)	1 (5.0)	1 (5.0)	1 (5.0)	11 (55.0)

Values are mean ± SD with both groups pooled unless stated. F and p are from one-way ANOVA across school levels. The interaction p value tests whether the between-group difference in change differs by school level (two-way ANOVA on change scores). All analyses in this table are exploratory and unadjusted for multiplicity. Knee symptoms differed by school level at baseline and week 12 (Fisher–Freeman–Halton exact test, $p = 0.001$ and $p < 0.001$).
